# Children with Poor Motor Skills Have Lower Health-Related Fitness Compared to Typically Developing Children

**DOI:** 10.3390/children8100867

**Published:** 2021-09-29

**Authors:** Marisja Denysschen, Dané Coetzee, Bouwien C. M. Smits-Engelsman

**Affiliations:** 1 Physical Activity, Sport and Recreation (PhASRec), Focus Area, Human Movement Sciences, Faculty of Health Science, North-West University, Private Bag X6001, Potchefstroom 2520, South Africa; marisja.denysschen@gmail.com (M.D.); bouwienengelsman@icloud.com (B.C.M.S.-E.); 2 Department of Health and Rehabilitation Sciences, University of Cape Town, Rondebosch, Cape Town 7700, South Africa

**Keywords:** developmental coordination disorder (DCD), health-related fitness, obesity, fat percentage, body mass index, waist circumference, children

## Abstract

Most of the current empirical evidence regarding the relationship between health-related fitness and level of motor performance is based on children from high-income countries. Yet, children from low-resource areas may have fewer opportunities to develop their fitness skills. The aim of the study was to determine if South African children from both low- and middle-income areas scoring below the 16th percentile on the Movement Assessment Battery for Children-2 (probable-Developmental Coordination Disorder (p-DCD)) have lower health-related fitness levels than typically developing (TD) children. We hypothesized that children with p-DCD would have lower overall health-related fitness than TD children. A sample of 146 participants aged 10 to 11 (10.05 years (SD = 0.41)) was collected from schools in the North West Province of South Africa, on the basis of their poverty classification. Children were tested for anaerobic capacity and strength using the Bruininks–Oseretsky test of motor proficiency second edition (BOT-2) and aerobic capacity using the Progressive Aerobic Cardiovascular Endurance Run (PACER). Body composition was evaluated using body mass index corrected for age and sex (BMI-z), body fat (BF), and waist circumference. The data was analyzed using Spearman correlations and chi-squared tests. Statistically significant differences (*p* < 0.05) were found between groups for running and agility, strength, and aerobic capacity. No significant differences were found between p-DCD and TD groups in terms of body mass (36.1 kg vs. 33.3 kg), waist circumference (62.2 cm vs. 59.8 cm), BMI-z (19.7 vs. 17.6), and fat percentage (20.2 vs. 18.1%). Overweight and obesity prevalence was 15% in those with low socio-economic status (SES) and 27% in high SES. In conclusion, children with p-DCD had lower muscular strength, aerobic capacity, and endurance than TD children. Although it has been reported that children with p-DCD have a higher risk for overweight/obesity than TD children, this is not (yet) the case in 10–11-year-old children living in rural areas in South Africa (North West Province).

## 1. Introduction

Developmental coordination disorder (DCD) is a disorder that is characterized by difficulties in motor coordination and problems with the execution of activities such as running, writing, getting dressed, hopping, and catching objects [1]. The prevalence of DCD in school-aged children is currently being reported between 1.4% and 19%, which makes this one of the more common childhood disorders [2,3,4,5,6].

Children with low motor proficiency, such as those with DCD, are less likely to participate in physical activities at home, school, and the community, being often excluded from activities by their peers and tending to display a lower fitness level than typically developing (TD) children [7,8,9,10].

Many studies reported reduced health-related fitness in children with DCD measured by explosive power, strength, and endurance [11,12,13,14], as well as lowered VO_2_max scores and cardiorespiratory fitness [15,16,17,18,19]. Although most studies were conducted in high-income areas, studies in low-to-middle income areas also found that health-related fitness was lowered in children with poor motor proficiency [8,20], suggesting this phenomenon is rather omnipresent. Smits-Engelsman and Bonney [21] reported that children with probable-DCD (p-DCD) in low-resource areas of South Africa displayed significantly poorer aerobic fitness than children without DCD. This is especially concerning given the lack of formal physical education in many school systems in these areas, resulting in less opportunities for children to engage in physical activity, which could enhance their motor proficiency and health-related fitness.

An important aspect related to health is body composition. Various researchers reported that children with DCD had higher body mass index (BMI) scores, waist circumference, and body fat than TD children [22,23,24,25]. In addition, one study found that children with p-DCD showed higher waist circumference, which indicates significantly higher levels of abdominal obesity [26]. A larger waist circumference can lead to a greater prevalence of health risks such as cardiovascular disease, insulin resistance, musculoskeletal disorders, some forms of cancer, and disability associated with increased weight [26,27,28,29]. Furthermore, only a few studies have examined the diverse aspects of health-related fitness among the same children with different levels of motor proficiency [30,31]. Additionally, most of the studies in the literature investigated BMI, with only a few studies incorporating waist circumference and fat percentage. Hence, despite the apparent solid evidence base, research on the interplay between body composition, fitness, and motor skills is sparse among children from low- and middle-income areas. By conducting research in low-to-middle income areas of South Africa, we can produce findings that can be generalized to other developing nations.

In conclusion, most of the literature suggests low motor proficiency negatively affects health-related fitness components and that a dearth of research exists in knowledge about health-related fitness in children with and without DCD living in low-resource areas. Therefore, the purpose of this study was to determine differences in health-related fitness between children with low motor proficiency and TD children. On the basis of the findings in the literature, we hypothesized finding lower aerobic capacity, strength and muscular endurance, anaerobic capacity, and agility, and higher BMI, body fat percentage, and waist circumference in the group with lower motor performance scores compared to typically developing children. Moreover, moderate correlations were expected between the factors constituting health-related fitness (aerobic capacity, strength and muscular endurance, anaerobic capacity, and agility) and body composition (BMI, body fat percentage, and waist circumference).

## 2. Materials and Methods

### 2.1. Study Design

The study formed part of the North-West Child Health Integrated with Learning and Development (NW-CHILD) study. The current study was a secondary analysis of the NW-CHILD study and is based on a cross-sectional study design. The participants were chosen by use of a stratified random sampling method. To determine the representative sample, we obtained a list of all schools in the North West Province from the Department of Basic Education. The list consisted of eight school districts, each with 12 to 22 zones and approximately 20 schools (minimum 12, maximum 47) per zone. From the list, four zones and 20 schools were randomly selected in terms of population and school status. Schools in South Arica are divided into five quintiles on the basis their poverty classification by the Department of Basic Education. The quintiles range from quintile 1 (low SES) to quintile 5 (high SES). The five quintiles were split into two groups, wherein quintiles 1 to 3 represented the low SES group (these children are on a food program) and quintiles 4 and 5 represented the high SES group. Five schools were chosen from each zone, and each school represented one of the quintiles. For the purposes of this study, only one cohort was used with participants in the grades 3 and 4, as this is when children begin to experience a decline in physical activity and overall well-being [32,33].

### 2.2. Participants

Ethical approval was obtained from the Health Research Ethics Committee (HREC) of the NWU for this sub-study (no. NWU-00939-19-A1). The population consisted of 219 participants from the Zeerust district, considered a predominantly rural area with both low- and middle-income areas. The participants had an average age of 10.05 years (SD = 0.41) with ages ranging from between 8.6 and 10.6 years. From the 219 children, only 146 of the data sets were complete and were used for the study. Of these, 61% were categorized as low (quintiles 1–3) and 39% as high SES background (quintiles 4–5). The data collection was performed by post-graduate students with a degree in Human Movement Science specializing in Kinderkinetics. Translators were used to correctly explain what was expected of the participants if the participants did not speak Afrikaans or English. Only participants who had written parental permission and who gave assent themselves were included in the study. Children who had neurological or intellectual impairments were excluded from the study. The Movement Assessment Battery for Children second edition (MABC-2) was used to identify participants with standard scores below the 16th percentile. Since not all DSM-5 criteria for DCD were checked in this study, participants with a score at or below the 16th percentile are referred to as the p-DCD group [34]. The measurements were performed in a safe environment, and each procedure was explained in detail to the participant.

### 2.3. Measurements

#### 2.3.1. Movement Assessment Battery for Children, Second Edition (MABC-2)

The Movement Assessment Battery for Children, second edition (MABC-2) is a test that can be used to identify children between the ages of 3 and 16 years with impaired motor function [35]. The test consists of three age bands: age band one (3 to 6 years), age band two (7 to 10 years), and age band three (11 to 16 years). For this study, only age band two was used. There are eight subtests in each age band, with three categories, namely, manual dexterity, aiming and catching, and balance. The raw score was converted to a standard score and percentile. Percentile scores of five or less indicate severe motor problems, while a score between 5 and 16 suggests the child is at risk of having movement difficulties. A percentile ranking above 16 indicates performance in the normal range of the tested motor skills. The test is a reliable measuring instrument with a test–retest reliability of ∝0.88–0.99 for the component scores and 0.97 for the total test scores [36].

#### 2.3.2. Progressive Aerobic Cardiovascular Endurance Run (PACER)

The PACER (also known as a 20 m shuttle run test) was used to measure aerobic capacity. This test consists of a 20 m distance that is run back and forth to the sound of beep, with an increase in intensity as the multi-stage fitness test progresses. The aim of the test was for children to run for as long as possible and to ensure they touch the line when the beep sounds. Children were required to continue until they could not run anymore or until they failed to reach the line twice. The number of laps completed was then recorded and used to determine aerobic capacity. Maximum oxygen uptake (VO_2_max) is the best method for determining aerobic capacity. This is measured by use of calculations specifically designed for the PACER and is based on age, sex, and laps completed. VO_2_max was calculated using the following formula: 31.025 + 3.238 ∗ (SpeedShuttle) − 3.248 ∗ (Age) + 0.1536 ∗ (SpeedShuttle ∗ Age) [37]. The PACER test is a reliable test in children [38].

#### 2.3.3. Bruininks–Oseretsky Test of Motor Proficiency, Second Edition (BOT-2)

The Bruininks–Oseretsky Test of Motor Proficiency, second edition (BOT-2) is a normative test that measures motor skills in children from 4 to 21 years of age [39]. For this study, only the sub-test strength and the sub-test running speed and agility were used. Each of these sub-tests has five items. The sub-test strength consists of standing long jump, knee push-up, sit-ups, wall sit, and the v-up. This sub-test was used to determine strength and muscular endurance. The sub-test running speed and agility was used to measure anaerobic capacity and agility, and includes the following activities: 15 m shuttle run, stepping sideways over a balance beam, one-legged stationary hop, one-legged side hop, two-legged side hop. The raw score was converted to a scale score per subtest and composite standard score for strength and agility, which was used to determine the child’s performance in the test. The BOT-2 has an internal consistency score of *r* ≈ 0.81 to r ≈ 0.88 for the subtests and *r* ≈ 0.87–0.88 for the composites, as well as a test–retest reliability of *r* ≈ 0.88–0.99, which makes this a reliable measurement tool [40].

#### 2.3.4. Anthropometric Measurements

The anthropometric measurements used to measure body composition in this study form part of the standard protocol as set out by the International Society for the Advancement of Kinanthropometry [41]. The following measurements were taken: stature in centimeter (cm), body mass in kilogram (kg), and waist circumference (cm). Stature was measured to the nearest 0.1 cm with the use of a portable stadiometer. Body mass was measured to the nearest 0.1 kg with the use of the Omron BF511 scale. BMI-*z* ((kg/height (m)^2^) was calculated by using stature and body mass and adapted for age. International age and gender-specific cut-off points were used to determine prevalence of obesity and overweight [42].

Waist circumference was measured at the smallest point between the lower costal rib (10th rib) and the iliac crest. Participants were in a standing position during the measurement, and a standard measuring tape was used (0.1 mm). The boys and girls were measured separately by someone of the same sex, where possible.

Fat percentage was measured by the subscapular, triceps, and calf skinfolds. The skinfolds were measured with Harpenden skinfold calipers. Each skinfold (triceps, subscapular, and calf) was taken twice, and the average of the two measurements was used. Item scores and total skinfolds score were used for the analysis.

### 2.4. Statistical Analysis

Statistical analyses were performed with SPSS 27.0. Demographic data (age, sex, SES) were used to describe the sample. Descriptive statistics (mean, standard deviation, median, maximum and minimum) was used for comparison between the two groups. Additionally, frequencies of the classifications for motor proficiency, fitness, and BMI were reported. Outcome measures were first checked for normality with the Shapiro–Wilk test. Given the distribution of the data, differences in health-related fitness outcomes between groups were investigated with the nonparametric Mann–Whitney *U* test. Eta squared effect sizes were calculated using the squared z-value of the Mann–Whitney *U* tests divided by the number of subjects minus 1, being defined as small (η^2^ = 0.01), medium (η^2^ = 0.06), and large (η^2^ = 0.14) effects [43]. For comparability to other studies, we added estimates of Cohen’s *d* for the significant differences between groups. Spearman correlations were calculated between the PACER, running speed and agility, strength, and body composition outcomes. Differences in classification frequency for motor proficiency groups (TD/p-DCD) and SES (high/low) groups were tested for the fitness and body composition outcomes using cross tabulation. The chi-squared test was used to compare the frequency between the two groups. Significance was set at *p* < 0.05.

## 3. Results

### 3.1. Motor Performance

The results indicated that from the 146 participants, 37 scored at or below the 16th percentile of the MABC-2 and were classified p-DCD. As seen in Table 1, 25% were assigned to the p-DCD group, while 75% of the participants were in the TD group.

The descriptive statistics of the results revealed that the boy/girl ratio was 1:1. Sex was equally distributed between TD and p-DCD groups. No significant differences were found between TD and p-DCD groups in terms of age, sex, or SES. Table 1 shows the distribution among children with and without p-DCD in the different BMI categories. One child that scored low on the MABC-2 was morbidly obese (BMI-z: 61.2).

### 3.2. Group Differences Regarding the Health-Related Fitness

Table 2 shows the results in aerobic and anaerobic capacity, and muscular strength between children with p-DCD and TD children. Mean, standard deviation, median, minimum and maximum values, and interquartile range are given in Table 3.

### 3.3. Aerobic Capacity

The PACER shuttle run test was used to determine the aerobic fitness and VO_2_max scores of the participants. Data for one child was missing. The results (Table 1) indicated statistically significant differences for PACER laps (*p* = 0.008), running speed (*p* = 0.018), and VO_2_max (*p* = 0.01) between children with low motor proficiency and TD children. Of the TD children, 47.7% (*n* = 52) scored below the 20th percentile, while this was 66.7% (*n* = 24) for the p-DCD group. In terms of international norms, we found that 52.4% of the total group scored below the 20th percentile, indicating a low level of aerobic fitness in this group of children [44].

### 3.4. Anaerobic Capacity and Agility

The running speed and agility sub-test of the BOT-2 was significantly different (*p* = 0.001) between groups with a medium effect size (Table 2 and Table 3). In all five running speed and agility test items, the TD children outperformed the children with p-DCD.

### 3.5. Strength and Muscular Endurance

The strength measures of the BOT-2 were used to test for strength and muscular endurance (Table 2 and Table 3). There were significant differences between the children with low motor proficiency and TD children and a high effect size for the total strength scale score (*p* = 0.001, medium effect size) and for three of the five items scores. Statistically significant differences were found for push-ups (*p* = 0.005), wall sit (*p* = 0.004), and v-up (*p* = 0.001) between children with p-DCD and TD children with small effect sizes.

For the combined strength and agility scale score, an age-corrected standard score or composite score is available. According to the United States of America (USA) norms, 5% of the TD children scored below the 20th percentile, and this was 32.4% for the children with p-DCD. The mean percentage of the groups was 12.3% below the 20th percentile, indicating relatively good levels of strength and agility.

### 3.6. Body Composition

No statistical differences were found between the children with p-DCD and TD children on any of the body composition outcomes, indicating that the groups were similar in terms of weight, BMI, waist circumference, and body fat scores (see Table 2 and Table 3).

Table 4 shows that distribution among children from the lower and higher SES group was different in BMI categories (chi^2^ 8.07, *p* = 0.045; see also Table 1). Of the children on the food program, 12% were underweight, while this was 2% for the children in the higher SES group. For overweight and obese classification, these were 15% and 27%, respectively.

BOT-2 and PACER outcomes were associated with body composition values (see Table 5). Lower fat percentage was associated with better fitness outcome, most clearly for the PACER (*r* = 0.39). SES was not related to aerobic and anaerobic capacity or muscular strength, but was to measures of body composition (*r* = 0.31–0.43).

## 4. Discussion

This study compared children with p-DCD to TD children. The results indicated statistically significant differences in health-related fitness between children with p-DCD and TD children (Table 4). Children with scores at or below the 16th percentile on the MABC-2 had overall lower scores in terms of running, agility, and strength subtests of the BOT-2 than their TD counterparts with medium effect sizes. In addition, a statistically significant difference was found for aerobic capacity. Hence, children with poor motor skills are more likely to have lower aerobic and anaerobic capacity and muscular strength compared to TD children. These findings imply that motor skills, cardiovascular fitness, muscular strength, and muscular endurance are intertwined.

Importantly, no differences were found between children with p-DCD and TD children in any of the body composition outcomes. The researchers viewed this result as indicating that low motor skills lead to lowered participation in physical activity, which in turn leads to a further decline in health-related fitness and motor skills. If this spiral continues and participation in physical activity keeps declining, this can lead to health effects such as overweight and obesity. However, it looks as though this negative spiral is not present in the current group.

The aerobic and anaerobic capacity and muscular strength results of the current study align with the results of various researchers in other countries [11,12,18]. For example, Hiraga et al. [8] reported that children with low motor proficiency in Brazil had significantly lower performance in cardiorespiratory fitness, explosive power, muscle strength, and endurance.

The fact that children with p-DCD had lower running speed and are less agile than their TD counterparts concurs with Smits-Engelsman et al. [31], who found that children with low motor proficiency had lower performance in all fitness measures including agility. Various other studies found that children with low motor proficiency had lowered performance in agility measures [21,31,45].

Moreover, lowered strength and power was reported in studies from South Africa [46] as well as in Tunisia [47]. In addition, several studies reported lower muscle strength among children with p-DCD [11,16,19]. Importantly, children with p-DCD had less gains in muscle strength with growth in height than TD children [48], which might indicate that the gap between children with p-DCD and their peers becomes larger when they grow older.

The current study confirms that children with low motor proficiency in a rural area of South Africa (North West Province) differ significantly from TD children in health-related fitness tests. Moreover, children in rural (also seen as low SES area including quintile 1–3) areas could have fewer opportunities to engage in organized physical activity and sport. This can negatively affect the development of skills specific to participation in these activities. Children in low- and middle-income countries are also more likely to be malnourished. The relationship between gross motor competence and BMI needs to be studied, further taking factors such as SES, physical inactivity, malnutrition, and physical fitness into account [49].

The results of this study are comparable in different SES settings and add to the external validity of the findings. The differences reported in different studies regarding fitness may have been due to the differences in neurological and physiological constraints between poorly coordinated and TD children. Given the large differences between the countries and children in the different samples, including this study, these universal findings on many different field tests point towards the coordination and motor planning difficulties to be scrutinized as an important underlying cause, as seen in this study as well. Utesch and colleagues [50] concluded the overlap in content between measures of motor competence and physical fitness warrants further investigation into the content and construct validity of the assessment tools. Ferguson et al. [51] suggested fitness tests may measure different constructs in children with DCD compared to TD children because it is very hard to take the coordination component out of a fitness field test. As an example, Cairney et al. [22] examined the concurrent validity of the PACER sprint test. The authors found significantly lower correlations between the PACER and the ergonometric test among children with MABC-2 scores at or below the fifth percentile and advised caution with the use of the shuttle run for children with severe motor coordination difficulties. Reasons for the low correlations could be due to the pacing and motor planning inherent to the PACER sprint test. Another example of the impact of coordination was reported by Ferguson et al. [51], who found no differences among groups in strength when using a single joint hand-held dynamometer but found significantly lowered performances in the *p* = DCD group for the functional strength measure (FSM), where multi-joint activities are tested. The authors furthermore concluded that timing and coordination of the movement pattern may be a greater influence than strength in these functional activities in which explosive power and repetitive movements (endurance) are important factors [51]. Supporting this, a review study found an increasing relationship between motor competence and physical fitness as children aged [50]. The study also indicated that neuromuscular function plays a direct role in the development of motor competence and physical fitness [50]. Our findings in this specific group, further strengthens the theory that coordination plays a significant role in agility, power, and cardiorespiratory field tests in children with DCD.

The current study found no statistically significant differences in BMI between children with p-DCD and TD children. This is in concurrence with some studies that found no statistical difference in BMI among children with DCD and TD children [52,53]. In contrast, other studies found differences in BMI between children with DCD and TD children [24,51]. One South African study found that children with DCD in low-income areas around Cape Town had higher BMI, weight, and waist circumference levels than children without DCD [51]. It seems that the relation between body fat percentage, waist circumference, and motor skill level is inconclusive. Only one study reported no significant differences in body fat percentage between children with p-DCD and TD children in Taiwan [19]. This is in contrast to studies that reported higher levels of waist circumference and body fat percentage in children with p-DCD [10,22,23,24]. A possible explanation of why the results of the current study differ with the literature is the specific selection of children. A large number of children in South Africa are on meal plans, which may explain why obesity is not yet a concern in young children with p-DCD [54,55,56]. However, body composition of these children may increase due to the lack of formal physical education that is not well implemented in the South African school system and the lack of the opportunity to participate in afterschool sport programs [57,58,59,60]. Only a small percentage of South African children actively engage in physical activity and structured sports [60]. In developed countries, participation in physical education is mandatory while at school [61,62]. In developed countries, it might therefore be more likely to see the effect of withdrawal from physical activities by children with DCD, while in South Africa, not taking part in structured physical activity occurs more extensively.

The strength of the study is that it is a representative sample of children from both lower- (quintile 1–3) and middle (quintile 4–5)-income areas. The study also includes the use of different tests to determine health-related fitness. This is furthermore one of a few South African studies that investigated children with p-DCD in rural/low-income areas (North West Province), with earlier studies conducted in the Free State and Western Cape Provinces. Another strength is the use of multiple measurements for body composition, namely, waist circumference, body fat percentage, and BMI-*z* scores to measure the adiposity levels of the participants more accurately.

A limitation of the study is the different sample sizes of the groups, but this is inherent to the diversity of low-and-middle income and quintile schools inherent to the South African context. The study only tested one age group and could not test for differences in ages. Another limitation of the study is that flexibility and an objective measure for participation in physical activity were not included. Recommendations for future studies is to use a longitudinal design to investigate changes in children over time. Within this longitudinal study, correlations between physical activity, sedentary behavior, perceived motor skill competence, and all components of health-related fitness can be explored to study the differences that may occur during development. The impact of coordination and motor planning in strength and fitness tests should be further explored in future studies. Future studies can also investigate the differences between boys and girls who have p-DCD and do not. Cultural influences can also be explored given the diverse ethnicities which presents in the South African context.

## 5. Conclusions

Children with low motor proficiency had poorer results in the following fitness components: aerobic capacity, muscular strength, and endurance, when compared to TD children. This indicates that children with low motor proficiency in low-to-middle-income areas also had lowered health-related fitness than TD children, indicative of the coordination aspect to be the underlying cause. In summary, the results are in line with the current literature in which children with low motor proficiency have lower health-related fitness than their TD peers. However, the study found that children with low motor proficiency did not have higher levels of body weight, waist circumference, and body fat percentage than TD children. This may change when children grow older due to a low participation in physical activities. The study also found no differences in prevalence of p-DCD between boys and girls. In conclusion, results of current study emphasize the importance of testing for health-related fitness in children suspected of neurodevelopmental disorders. This information could be useful to ameliorate the negative impact on health-related fitness caused by poor motor skills and inactivity.

## Figures and Tables

**Table 1:** Frequency of participants in different BMI, PACER, and BOT-2 classifications, and statistics.

	TD		p-DCD			
	Frequency	Percent	Frequency	Percent	Chi-Square	Odds Ratio
Sex						
Boys	55	50.5	18	48.6	0.52, *p* = 0.82	n.a.
Girls	54	49.5	19	51.4		
PACER classification						
At or below 20th SR	52	47.7	24	64.9	3.90, *p* = 0.048 *	1.57 (0.96–2.58)
Above 20th SR	57	52.3	12	32.4		
BOT-2 strength and agility						
At and below 20th percentile	6	5.5	12	32.4	18.53, *p* = 0.001 *	1.40 (1.11–1.76)
Above 20th percentile	103	94.5	25	67.6		
SES						
Low (quintile 1–3)	65	59.6	24	64.9	0.32, *p* = 0.57	n.a.
High (quintile 4–5)	44	40.4	13	35.1		
Total	109	100	37	100		
BMI classification						
Underweight	9	8.3	3	8.1	8.07, *p* = 0.45	n.a.
Normal weight	80	73.4	25	67.6		
Overweight	13	11.9	4	10.8		
Obese	7	6.4	5	13.5		

Note. *n*: number of participants; p-DCD: probable developmental coordination disorder; TD: typically developing; n.a.: not applicable; *: *p* < 0.05.

**Table 2:** Statistics for the comparison between motor proficiency groups on health-related fitness (aerobic, anaerobic capacity and agility, strength and muscular endurance, and body composition).

Variable	Total (*n*)	U-Value	z-Value	*p*-Value (2-Sided Test)	Eta Squared	Cohen’s *d*
Aerobic capacity						
PACER (laps)	145	1380	2.66	0.008 *	0.04	0.41 •
Shuttle run stage	145	1463	2.36	0.018 *	0.03	0.35 •
VO_2_ max	145	1420	2.50	0.012 *	0.04	0.41 •
Anaerobic capacity and agility						
Shuttle run	146	2617	2.70	0.007 *	0.05	0.46 •
Stepping sideways	146	1200.5	3.67	<0.001 *	0.09	0.63 ^▲^
One legged stationary hop	146	1567.5	2.02	0.043 *	0.02	0.29 •
One legged side hop	146	1295	3.25	0.001 *	0.07	0.55 ^▲^
Two-legged side hop	146	1173.5	3.80	<0.001 *	0.09	0.63 ^▲^
Running speed and agility scale score	146	1067.5	4.16	<0.001 *	0.11	0.70 ^▲^
Strength and muscular endurance						
Standing long jump	146	1597	1.88	0.059	0.02	0.29 •
Push-ups	146	1388	2.83	0.005 *	0.05	0.46 •
Sit-ups	146	1617	1.80	0.072	0.02	0.29 •
Wall sit	146	1528	2.84	0.004 *	0.05	0.46 •
V-up	146	1350	3.48	0.001 *	0.08	0.59 ^▲^
Strength scale score	146	1271	3.37	0.001 *	0.07	0.55 ^▲^
Composite score of strength and agility	146	1080	4.22	<0.001 *	0.12	0.74 ^▲^
Body composition						
Height (cm)	146	1889	0.57	0.566		
Weight (kg)	146	1930	0.39	0.695		
BMI	146	2070	0.24	0.810		
BMI-z	146	1934	0.37	0.711		
Waist circumference (cm)	146	2041	0.11	0.912		
Fat percentage	146	2089	0.32	0.744		
Skinfolds						
Subscapular skinfold	146	2253	1.06	0.287		
Triceps skinfold	146	2174	0.71	0.477		
Calf skinfold	146	2158	0.63	0.523		

Note. *: *p* < 0.05; Cohan’s *d* = 0.2 • small effect size; ▲ d = 0.5 medium effect size.

**Table 3:** Mean, standard deviation, median, minimum and maximum values, and interquartile range for health-related fitness (aerobic and anaerobic capacity, muscular strength, and body composition) for the two groups.

Description	*n*	Mean	SD	Median	Min	Max	Interquartile Range
Aerobic capacity							
PACER laps							
p-DCD	36	43.7	3	43.3	4	48	11.7
TD	109	21.6	12.1	20	5	74	18.5
Group	145	20.4	12	18	4	74	17
Shuttle run stage							
p-DCD	36	3	1.3	2.5	1.4	6.7	2.5
TD	109	3.6	1.4	3.5	1.5	9.2	2.2
Group	145	3.5	1.4	3.3	1.4	9.2	2.1
VO_2_max							
p-DCD	36	43.7	3	43.3	39.1	51	4.1
TD	109	44.9	3.2	43.8	33.4	53.4	2.9
Group	145	44.6	3.2	43.8	33.4	53.4	3.3
Anaerobic capacity and agility							
Shuttle run							
p-DCD	37	9.5	1.2	9.3	7.3	12	1.1
TD	109	9	0.7	8.9	7.3	11	1
Group	146	9.1	0.9	9.1	7.3	12	1.1
Stepping sideways							
p-DCD	37	31.9	9	32	8	55	12
TD	109	37.7	7.9	38	11	58	11
Group	146	36.3	8.6	37	8	58	12
One legged stationary hop							
p-DCD	37	38	7.1	38	23	50	9
TD	109	41.3	7.6	41	25	60	12
Group	146	40.5	7.6	40	23	60	10
One legged side hop							
p-DCD	37	20.1	7	19	10	32	12
TD	109	24.8	6.5	25	11	42	10
Group	146	23.6	6.9	24	10	42	10
Two-legged side hop							
p-DCD	37	24.6	5.7	25	10	35	7
TD	109	39.1	5.3	29	16	40	8
Group	146	28	5.8	28.5	10	40	30
Running speed and agility scale score							
p-DCD	37	14.9	3.4	15	10	22	6
TD	109	17.6	2.7	18	11	23	5
Group	146	16.9	3.1	17	10	23	4
Strength and muscular endurance							
Standing long jump							
p-DCD	37	38.4	7.8	40.7	17.8	49.6	10.2
TD	109	42.1	8.2	41.6	8.3	64.3	9.8
Group	146	41.2	8.2	41.1	8.3	64.3	9.8
Push-ups							
p-DCD	37	14.8	5.5	15	0	25	7
TD	109	18.2	5.8	18	3	30	9
Group	146	17.4	5.9	17	0	30	8
Sit-ups							
p-DCD	37	16.5	5.4	17	0	23	6
TD	109	18.9	6	19	5	50	6
Group	146	18.3	5.9	18	0	50	6
Wall sit							
p-DCD	37	47.7	16.7	60	9	60	28
TD	109	56.1	9.2	60	17	60	0
Group	146	54	12.1	60	9	60	4
V-up							
p-DCD	37	40.5	21.9	50	0	60	36
TD	109	53	13.1	60	0	60	11
Group	146	49.8	16.6	60	0	60	15
Strength scale score							
p-DCD	37	13.9	3.6	15	5	19	6
TD	109	16.6	2.9	16	9	24	4
Group	146	15.9	3.3	16	5	24	4
Composite Score Strength and agility							
p-DCD	37	48.5	7.6	50	22	61	12
TD	109	55	5.7	55	42	67	9
Group	146	53.4	6.8	54	33	67	9
Body composition							
Height (cm)							
p-DCD	37	137	7.6	134.4	123.8	155.1	11.2
TD	109	137.2	7.7	136.4	123.2	161	8.8
Group	146	137.2	7	136.5	123.2	161	9.2
Weight (kg)							
p-DCD	37	35	9.1	30.1	21	59.9	10
TD	109	33.2	7.7	32.1	22.2	59.9	9.6
Group	146	33.1	8	31.6	21	59.9	9.6
BMI-*z* scores							
p-DCD	37	17.3	3.7	17.1	12.2	27.7	3
TD	109	17.4	2.9	16.7	10	29.7	2.9
Group	146	17.4	3.1	16.7	10	29.7	2.9
Waist circumference (cm)							
p-DCD	37	59.8	7.5	57.8	46	81.5	7.2
TD	109	59.8	6.6	58.2	51	83	6.9
Group	146	59.8	6.8	58.1	46	83	6.9
Fat percentage							
p-DCD	37	18.8	8.2	17.3	9	41.6	7.7
TD	109	18.1	7.3	17.8	7.4	46.6	9.7
Group	146	18.2	7.5	17.6	7.4	46.6	8.6
Subscapular skinfold							
p-DCD	37	9.1	6.7	6.5	3.2	30.7	3.8
TD	109	7.8	4.6	6.5	3.5	31	3
Group	146	8.1	5.2	6.5	3.2	31	3.5
Triceps skinfolds							
p-DCD	37	11.6	5.7	9.5	5.2	27.7	8.1
TD	109	11.1	5.4	9.5	3.5	27.2	7.6
Group	146	11.2	5.5	9.5	3.5	27.7	7.3
Calf skinfolds							
p-DCD	37	13.4	7.5	11.5	5	35.2	7.6
TD	109	12.4	5.9	11.7	3.5	31.2	7.5
Group	146	12.8	6.6	11.7	3.5	35.2	7.3
Subscapular and triceps skinfolds sum							
p-DCD	37	20.8	12.3	16.5	9.5	58.5	10.2
TD	109	18.9	9.8	16.5	7.5	57.5	10.7
Group	146	19.4	10.5	16.5	7.5	58.5	10.5

Note. *n*: number; SD: standard deviation; Min: minimum; Max: maximum; TD: typical developing; p-DCD: probable DCD; BMI-*z***:** body mass index corrected for sex and age.

**Table 4:** Frequency table of participants regarding SES and BMI categories.

		p-DCD	TD	Total
SES		Low SES	High SES	Total
Underweight	*n* (%)	11 (12.4)	1 (1.8)	12 (8.3)
Normal weight	*n* (%)	65 (73)	40 (71.4)	105 (72.4)
Overweight	*n* (%)	9 (10.1)	8 (14.3)	17 (11.7)
Obese	*n* (%)	4 (4.5)	7 (12.5)	11 (7.6)
Total group	*n* (%)	89 (100)	56 (100)	145 (100)

Note. %: percentage; p-DCD: probable DCD; TD: typically developing; *n*: number of participants; SES: socio-economic status.

**Table 5:** Interrelation between health-related fitness outcomes and SES.

Spearman’s Rho *n* = 145	PACER	Running Speed and Agility	Strength Scale Score	Strength and Agility	Weight (kg)	BMI	Waist Circumference	Fat	SES
Pacer laps	1	−0.376 **	0.494 **	0.483 **	−0.216 **	−0.288 **	−0.255 **	−0.392 **	0.031
Running speed and agility	0.376 **	1	0.522 **	0.876 **	−0.125	−0.097	−0.062	−0.188 *	0.012
Strength (scale score)	0.494 **	0.522 **	1	0.839 **	−0.072	−0.066	−0.069	−0.305 **	0.154
Strength and agility composite score	0.483 **	0.876 **	0.839 **	1	−0.144	−0.113	−0.089	−0.275 **	0.064
Weight (kg)	−0.216 **	−0.125	−0.072	−0.144	1	0.894 **	0.854 **	0.665 **	0.430 **
BMI (z-score)	−2.88 **	−0.097	−0.066	−0.113	0.894 **	1	0.831 **	0.661 **	0.357 **
Waist circumference (cm)	−0.255 **	−0.062	−0.069	−0.089	0.854 **	0.831 **	1	0.591 **	0.304 **
Mean fat (%)	−0.392 **	−0.188 *	−0.305 **	−2.73 **	0.665 **	0.661 **	0.591 **	1	0.307 **
SES	0.031	0.012	0.154	0.064	0.430 **	0.357 **	0.304 **	0.307 **	1

Note. SES: socio-economic status; %: percentage; BMI: body mass index corrected for sex and age; * *p* = 0.1 small correlation; ** *p* > 0.2 large correlation.

## Data Availability

The dataset is the property of the North-West University under supervision of Anita E Pienaar. In this regard, A.E. Pienaar should be contacted if, for any reason, the data included in this paper need to be shared. A.E. Pienaar is the principal investigator of this study and gave permission for the data to be used.
